# Effect of general anesthesia on postoperative outcomes of transcatheter aortic valve implantation in patients: a systematic review and meta-analysis

**DOI:** 10.3389/fcvm.2026.1714689

**Published:** 2026-07-16

**Authors:** Guanzhu Li, Junchen He, Yating Yang, Jinhe Deng, Chaokun Zeng, Gaofeng Zhao, Min Zhong

**Affiliations:** Department of Anesthesiology, Guangdong Hospital of Traditional Chinese Medicine, The Second Affiliated Hospital of Guangzhou University of Chinese Medicine, Guangzhou, China

**Keywords:** 30-day postoperative mortality rate, general anesthesia, meta-analysis, postoperative outcomes, TAVI

## Abstract

**Objective:**

The optimal mode of anesthesia for patients undergoing transcatheter aortic valve implantation (TAVI) surgery has been controversial recently, especially with the popularization of TAVI in young and low-risk patients and reduced dependence on transesophageal echocardiography (TEE). Beyond general anesthesia (GA), there are more than one type of anesthetic methods such as regional anesthesia (RA), local anesthesia (LA), monitored anesthesia care (MAC), deep sedation (DS), conscious sedation (CS) et al. used on TAVI. The aim of this systematic review and meta-analysis was to evaluate the effects of general anesthesia on the prognosis of patients undergoing TAVI.

**Methods:**

The Cochrane Library, PubMed, Embase, and Medline databases were searched from their inception to May 2025. Literature was selected according to the inclusion and exclusion criteria, and the meta-analysis was completed using RevMan 5.3.

**Results:**

A total of eligible 38 literatures were enrolled, including 23,848 patients. The results of the meta-analysis showed that compared with the non-GA groups, the in-hospital mortality (RR = 1.99, 95%CI, 1.19–3.30, *P* = 0.008), incidence of postoperative pneumonia (RR = 2.39, 95%CI, 1.43–4.00, *P* = 0.0009), procedure time (MD = 20.22, 95%CI, 15.37–25.07, *P* < 0.00001), length of hospital stay (MD = 1.43, 95%CI, 1.10–1.76, *P* < 0.00001), and ICU stay (SMD = 1.91, 95%CI, 1.40–2.42, *P* < 0.00001) were all increased in the GA group. There were no significant differences between the groups in 30-day mortality (RR = 1.19, 95%CI, 0.97–1.47, *P* = 0.09), postoperative acute kidney injury (RR = 1.16, 95%CI, 0.90–1.50, *P* = 0.26), postoperative stroke (RR = 0.99, 95%CI, 0.80–1.22, *P* = 0.90), postoperative vascular complication (RR = 1.10, 95%CI, 0.92–1.33, *P* = 0.30), and postoperative myocardial infarction (RR = 1.12, 95%CI, 0.72–1.73, *P* = 0.61).

**Conclusion:**

GA not only increases in-hospital mortality and the incidence of postoperative pulmonary infections in patients undergoing TAVI but also prolongs the length of hospitalization and ICU stay. However, GA did not increase the incidence of postoperative acute kidney injury, stroke, myocardial infarction, or vascular complications, nor did it increase the 30-day postoperative mortality rate and long-term quality of life in patients. The choice of anesthesia for TAVI should be evaluated according to the patient's condition and surgical approaches to minimize adverse complications and mortality. Further RCTs are required to verify the most likely anesthetic choices for TAVI.

## Introduction

Aortic stenosis (AS) is common in older patients, especially when it is combined with angina, syncope or heart failure (1). In the past, surgical aortic valve replacement (SVAR) was the main treatment to reduce related symptoms and improve prognosis, and the overall mortality of most patients was low. However, many patients could not undergo SAVR due to advanced age, frailty, and other severe concomitant systemic diseases such as severe cardio-cerebrovascular complications. Transcatheter aortic valve implantation (TAVI) was invented more than 30 years ago. Since 2002, transcatheter aortic valve implantation (TAVI) has become an alternative option for elderly and high-risk patients (2). And TAVI has since become widespread adopted in clinical practice. Despite initially being reserved for high-risk patients who were not candidates for open heart surgery, use of TAVI is now expanding to low-risk patients as well as patients with failing bio-prostheses (3). TAVI has been increasingly chosen by clinicians because of its low trauma rate, quick postoperative recovery, and few complications. Given the cumulative encouraging clinical trial results, TAVI is supported to extend to low- and intermediate-risk patients.

The development of surgical techniques will inevitably lead to further optimization of anesthetic methods. Even in the early stage of the implementation of this technology, the surgical skills of the surgeons were not proficient. To ensure safety, the main anesthesia method chosen was tracheal intubation general anesthesia. As the surgical techniques and technologies accumulated and evolved, such as more precise imaging and locatable valve systems, the anesthesia strategies began to be optimized. Anesthesia doctors started to try various different anesthesia methods, such as deep sedation, anesthesia care under monitoring, local nerve block and local anesthesia, all with the aim of reducing postoperitive complications and accelerating recovery.

Regarding its practical implementation, the selection of anesthetic approaches for TAVI has been controversial, and there are no guidelines to follow. Some studies suggest that general anesthesia can facilitate TEE during TAVI surgery and can also quickly detect intraoperative complications; however, induction of general anesthesia also increases the risk of hemodynamic fluctuations in patients (4). Other studies suggest that non-general anesthesia, including regional anesthesia (RA), local anesthesia (LA), and MAC, can reduce hemodynamic fluctuations during induction, but increase the nervous mood and the incidence of respiratory depression and postoperative acute kidney injury (5). In recent years, cumulative clinical studies have compared and evaluated the effects of different anesthetic methods on the postoperative outcomes of patients undergoing TAVI. However, due to the differences and limitations of study endpoints, observation indexes, and sample sizes, there is still no consensus been reached yet. In this study, conducting a literature search and meta-analysis was used to compare the effect of general anesthesia and other anesthesia methods (including RA, LA, MAC et al.) on the postoperative outcomes of TAVI patients to provide further references for selecting the best anesthetic method for this operation.

## Methods

To perform this systematic literature review, we adopted the principles proposed in the Cochrane Handbook [Frohlich 2014] and followed the Preferred Reporting Items for Systematic Reviews and Meta-Analysis (PRISMA) statement [Moher 2010]. Registration number: CRD42023445797.

### Search strategy

Cochrane Library, PubMed, Embase, and Medline from inception to May 2025, as well as the accessibility of online citations in the literature. The literature was selected according to the inclusion and exclusion criteria. The keywords used in the search were: anesthesia, transcatheter aortic valve implantation (TAVI), postoperative outcome.

### Participants

Patients undergoing TAVI surgery without limitations on age and sex were selected for the meta-analysis.

### Intervention and controls

The patients in the GA group underwent general anesthesia, while those in the control group underwent non-general anesthesia (Non-GA), which included regional anesthesia (RA), local anesthesia (LA), monitored anesthesia care (MAC), deep sedation (DS), conscious sedation (CS), minimal conscious sedation (MCS), local anesthesia and sedation (LAS), sedation anesthesia (SA), and moderate sedation (MS).

### Outcomes

The primary outcomes were in-hospital mortality and 30-day mortality. The secondary outcomes were procedure time, length of hospital stay and ICU stay, and incidence of postoperative acute kidney injury, stroke, vascular complications, myocardial infarction, and postoperative pneumonia.

### Types of studies

Only randomized controlled trials on the effects of anesthesia on postoperative outcomes after TAVI surgery were considered eligible.

### Exclusion criteria

The exclusion criteria were as follows: (a) non-randomized trials; (b) non-general anesthesia in both groups; (c) reviews and case reports; and (d) animal experiments.

### Data extraction

Three evaluators independently read the literature, selected it according to the inclusion and exclusion criteria, and re-read the full text of articles that met the criteria for re-selection. Any disagreement between the three parties in the discussion was further referred to a fourth party (6) for their opinion. The following basic data were extracted: first author of the literature, age and number of participants, year of publication, intervention and control measures, anesthesia, in-hospital mortality and day 30 mortality, procedure time, length of hospital stay and ICU stay, incidence of postoperative acute kidney injury, stroke, vascular complications, myocardial infarction, and pneumonia.

### Risk of bias assessment of included studies

The quality of the included studies was independently evaluated by two reviewers according to the risk of bias assessment recommended by the Cochrane Evaluation Manual (6). The assessment comprised of the following seven domains: adequate sequence generation, blinding of outcome assessment, allocation concealment, blinding of participants and personnel, selective reporting, incomplete outcome data, and other biases. Each question was rated as yes (+), indicating a low risk of bias, unclear (?) which indicates a risk of bias, or no (−), which indicates a high risk of bias.

### Statistical analysis

The analysis was conducted using the RevMan 5.3. Risk ratio (RR) or odds ratio was used for dichotomous data. In each analysis, mean difference (MD) or standardized mean difference (SMD) was used for continuous data. We measured heterogeneity using *Q* statistics and the *I*^2^ index, according to the recommendations of the Cochrane Collaboration. *I*^2^ < 50% was considered no (*I*^2^ = 0) or low heterogeneity, and the outcome was pooled with a fixed-effects model; otherwise, a random-effects model was used. A meta-analysis was performed to calculate the RRs, SMDs, MDs, and 95% CIs using the Mantel-Haenszel statistical method. If zero events were reported for one group in comparison, a value of 0.5 was added to both groups for each study. Additionally, a subgroup analysis based on the different methods of anesthesia was performed. Sensitivity analyses were performed as required. The reporting and publication biases of the included studies were assessed by visually inspecting the funnel plot asymmetry (7).

## Results

### Study selection

**Figure 1:**
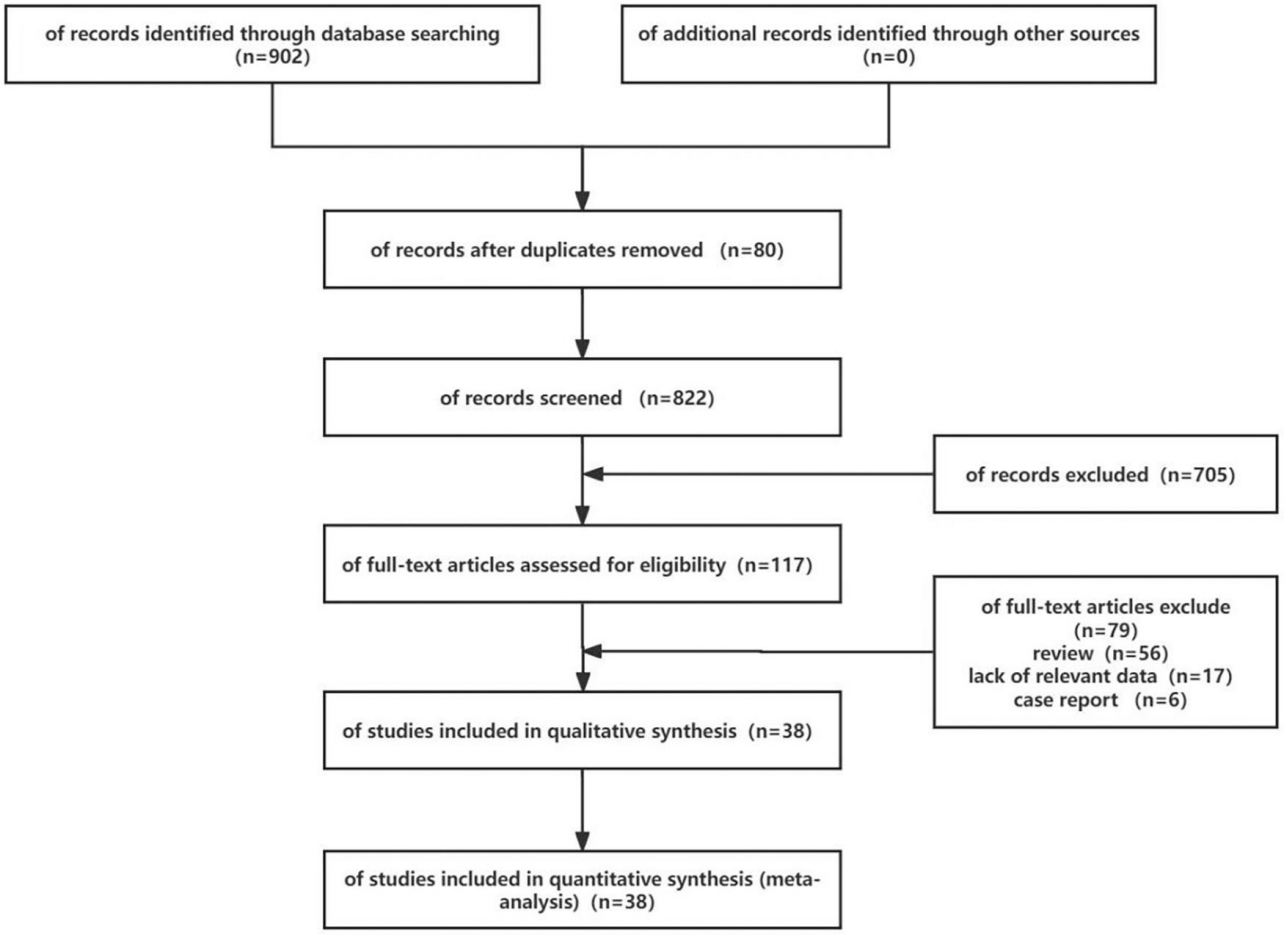
Flow chart of included and excluded studies.

In total, 902 articles were selected, including 773 from PubMed, 57 from the Cochrane Library, 15 from Embase, and 37 from MEDLINE. After reading the titles, abstracts, and full texts, 38 RCTs were included, all of which were published in English. These 38 articles included 23,848 patients. A flowchart of the included and excluded studies is shown in Figure 1.

### Characteristics and risk of bias assessment of included studies

**Figure 2:**
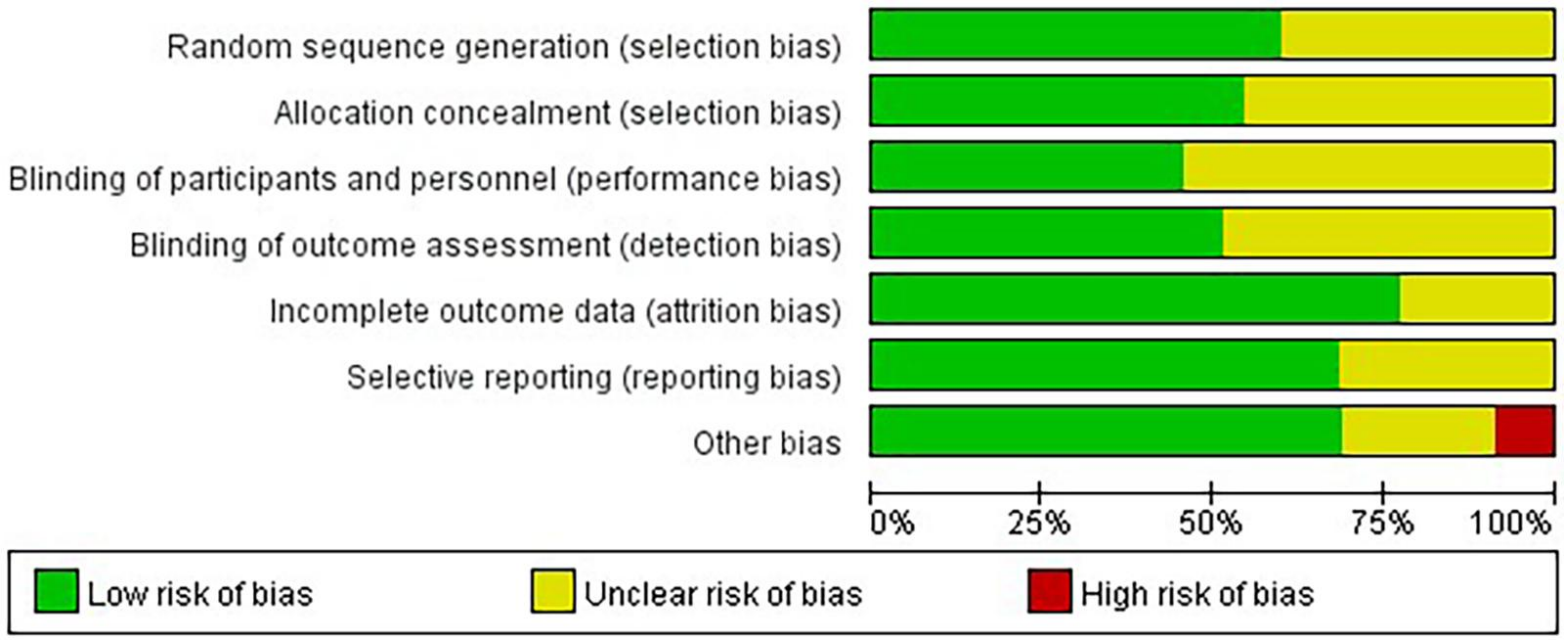
Risk of bias assessment of included studies.

General information, sample sizes, interventions, and outcome indicators of the included studies are shown in Table 1. The risk of bias assessment of the included studies is shown in Figure 2. The publication bias of the included studies is shown in Figure 13.

**Table 1:** Features of included studies.

Included studies	Sample (G/C)	Age (years) (G/C)	NYHA classes III-IV (G/C)	Interventions (G/C)	Outcome indicators
Domoto et al. (8)	25/19	84.3 ± 0.1	4/1	GA/RA	①③④⑤⑥⑦⑧⑨
		82.7 ± 1.3			
Sanders et al. (9)	38/41	81 (74–86)	/	GA/DS	①③④⑦⑧
		80 (73–86)			
Husser et al. (10)	2,624/2,624	81 ± 5	2,226/2,208	GA/LACS	①③④⑤⑦⑧⑨
		81 ± 6			
Dall'Ara et al. (11)	1,712/1,095	81.4 ± 7.1	1,361/828	GA/LA	①③④⑥⑦⑧⑨
		82.5 ± 7.0			
Dehédin et al. (12)	91/34	83 (78–75)	27/4	GA/LRA	②③④⑤⑥⑦⑧⑨
		83.5 (75–88)			
D'Errigo et al. (13)	310/310	82.0 ± 5.4	39/40	GA/MAC	②⑤⑥⑦⑧
		82.7 ± 5.8			
Stragier et al. (14)	85/93	81.7 (80.0–83.5)	69/64	GA/MAC	②③④⑤⑥⑦⑧⑨
		81.3 (79.6–83.1)			
Neumann et al. (15)	667/1,027	81.3 ± 6.6	55/97	GA/CS	②④⑤⑥⑦⑧⑨
		81.9 ± 6.72			
Liang et al. (16)	66/107	75.76 ± 6.17	61/89	GA/CS	②⑥⑦⑧⑨
		76.95 ± 5.84			
Motloch et al. (17)	33/41	83.4 ± 0.6	27/34	GA/LAPS	②③④⑤⑥⑦⑧
		82.6 ± 1.2			
Jabbar et al. (18)	145/71	80.2 ± 6.9	19/10	GA/LA	②③④⑦⑧⑨
		80.9 ± 6.8			
Pani et al. (19)	37/60	83 (78–89)	/	GA/MAC	②④⑤⑥⑦⑧⑨
		83 (77–88)			
Eskandari et al. (20)	1,816/427	81.2 ± 6.7	1,370/340	GA/Non-GA	②③④⑥⑧
		82.0 ± 6.8			
Musuku et al. (21)	148/151	81 (74–86)	/	GA/MAC	②③④⑥⑦
		83 (76–88)			
Abbett et al. (22)	159/159	80 ± 7.9	8/11	GA/MAC	②④⑦⑨⑩
		80 ± 8.12			
Aslan et al. (23)	42/30	77.8 ± 6.9	/	GA/CS	②③④⑤⑥⑦⑧⑨⑩
		78.1 ± 8.9			
Holmes et al. (24)	105/244	77.8/76.9	/	GA/MAC	②⑨
Zaouter et al. (25)	66/168	80.2 ± 7.5	39/95	GA/CS	⑥⑦⑨
		81.8 ± 8.4			
Miles et al. (26)	44/44	77.8 ± 7.8	36/38	GA/CS	①②③④⑥⑨
		81.5 ± 6.4			
Ben-Dor et al. (27)	22/70	83.7 ± 7.9	12/34	GA/MAC	①③④
		84.1 ± 5.1			
Herrmann et al. (28)	163/330	72.9 ± 5.54	62/92	GA/CS	②③④ ⑤
		73.6 ± 5.96			
Oguri et al. (29)	403/403	83.3 ± 6.9	311/307	GA/LA	②④⑤⑥⑦⑧⑨
		83.3 ± 7.8			
Thiele et al. (30)	220/218	81.4 ± 5.7	122/130	GA/CS	②⑥⑦⑨⑩
		81.8 ± 5.3			
Ahmad et al. (31)	224/194	81.8 ± 8.1	186/172	GA/CS	④⑤
		80.0 ± 8.8			
Attizzani et al. (32)	1,988/1,988	81.7 ± 7.7	1,315/1,310	GA/LA	①②⑦⑧⑨
		81.9 ± 7.6			
Mukdad et al. (33)	139/58	82.2 ± 12.4	57/34	GA/MS	③④⑤⑦⑩
		83.8 ± 9.2			
Goren et al. (34)	75/129	83 ± 5.5	31/48	GA/SA	①⑥⑧⑩
		83 ± 5.4			
Renner et al. (35)	107/93	82 ± 6.1	64/49	GA/CS	②③⑥⑦⑧⑨
		82 ± 6.4			
Kesimci et al. (36)	79/72	76.3 ± 8.6	/	GA/LAS	②③④⑤⑧
		77.4 ± 8.7			
Kiramijyan et al. (37)	66/467	81.3 ± 10.6	58/380	GA/MAC	①②④⑤⑥⑦⑧
		82.9 ± 7.6			
Lau et al. (38)	85/219	80.0 ± 10.6	65/129	GA/MCS	②④⑤⑥⑦⑧
		82.5 ± 7.3			
Téllez-Alarcón et al. (39)	76/82	78.1 (75.8–80.4)	43/39	GA/CS	①③④⑤⑥⑦⑧
		80.4 (78.9–91.9)			
Mosleh et al. (40)	154/154	80.31 ± 9.31	107/109	GA/CS	①②④⑤⑦
		82.06 ± 7.41			
Palermo et al. (41)	21/44	79.6 ± 10.9	/	GA/MAC	③④⑤
		85.4 ± 9.1			
Yamamoto et al. (42)	44/130	84.7 ± 7.0	28/86	GA/LACS	②③④⑤⑥⑦⑧⑨
		83.7 ± 7.1			
Izgi et al. (43)	40/119	79.1 ± 8.1	/	GA/CS	①②③④⑤⑥⑦
		75.7 ± 10.6			
Yılmaz et al. (44)	37/103	77.3 ± 9.3	/	GA/DS	③⑤
		78.9 ± 8.3			
Aslan et al. (23)	42/30	77.8 ± 6.9	/	GA/CS	②③④⑤⑥⑦⑧⑩
		78.1 ± 8.9			

G, general anesthesia group; C, control group; ① In-hospital mortality; ② day 30 mortality; ③ procedure time; ④ length of stay hospital; ⑤ ICU stay; ⑥ acute kidney injury; ⑦ stroke; ⑧ vascular complication; ⑨ myocardial infarction; ⑩ pneumonia. STS, society of thoracic surgeons; RA, regional anesthesia; LA, local anesthesia; DS, deep sedation; CS, conscious sedation; MAC, monitored anesthesia care; MCS, minimal conscious sedation; LAS, local anesthesia and sedation; SA, sedation anesthesia; LACS, local anesthesia or conscious sedation; MS, moderate sedation; LRA, local anesthesia; LAPS, local anesthesia plus mild sedation.

### Results of meta-analysis

#### In-hospital mortality and 30-day mortality

**Figure 3:**
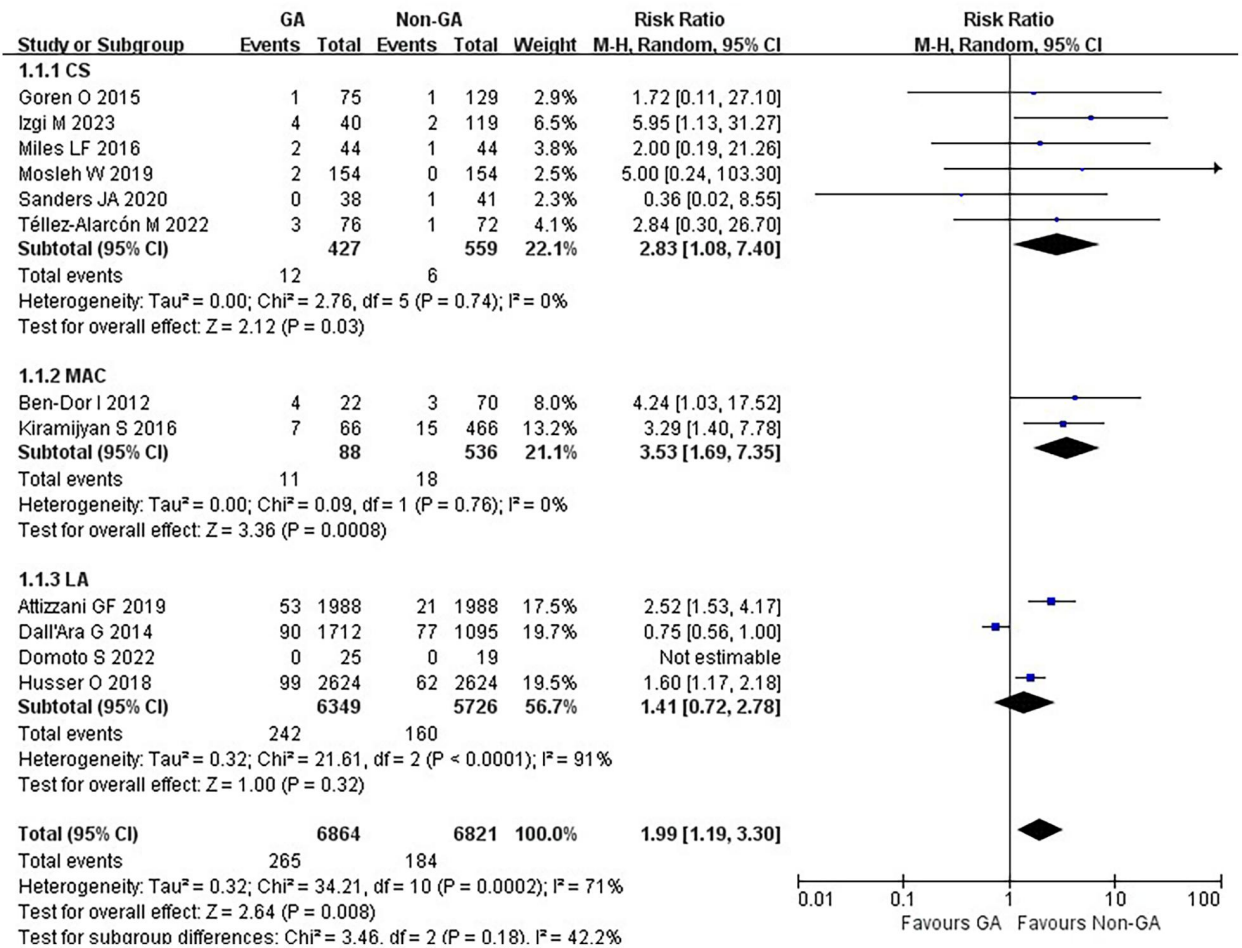
Meta-analysis of in-hospital mortality.

In-hospital mortality, based on 12 studies (8–11, 26, 27, 32, 34, 37, 39, 40, 43) showed that the in-hospital mortality in the GA group was higher than that in the control group (Figure 3), with a statistically significant difference (RR = 1.99, 95%CI, 1.19–3.30, *P* = 0.008). The subgroup analysis showed that there were no significant differences in in-hospital mortality between the GA group and LA subgroup (RR = 1.41, 95%CI, 0.72–2.78, *P* = 0.32). However, the in-hospital mortality in the monitored anesthesia care (MAC) subgroup (RR = 3.53, 95%CI, 1.69–7.35, *P* = 0.0008) and CS subgroup (RR = 2.83, 95%CI, 1.08–74, *P* = 0.03) was significantly lower.

**Figure 4:**
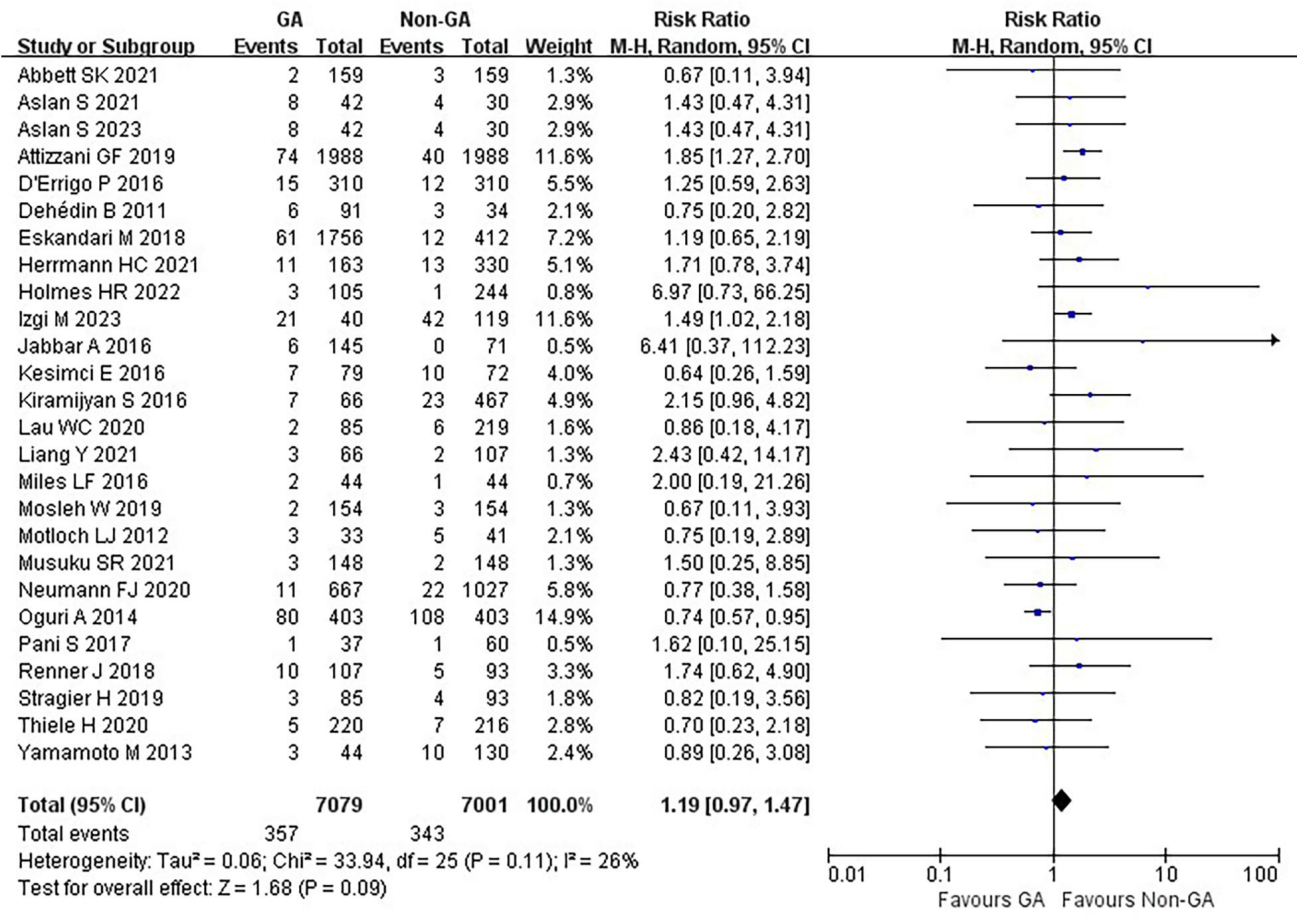
Meta-analysis of day 30 mortality.

The results of 30-day mortality, based on 26 studies (12–24, 26, 28–30, 32, 35–38, 40, 42, 43) showed that there was no significant difference in 30-day mortality between the general anesthesia group and the control group (RR = 1.19, 95%CI, 0.97–1.47, *P* = 0.09) (Figure 4).

#### Procedure time

**Figure 5:**
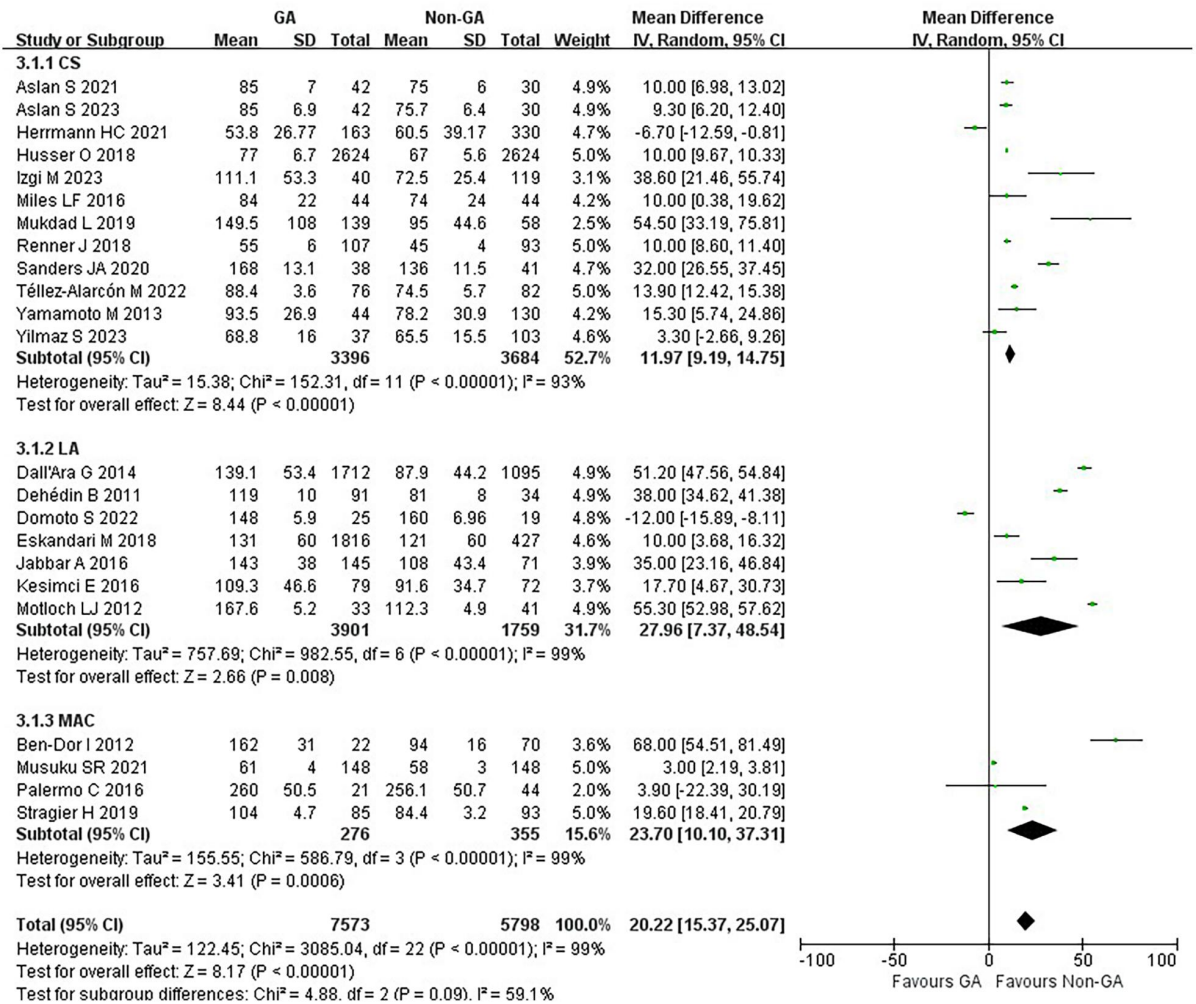
Meta-analysis of the procedure time.

The results of procedure time, based on 23 studies (8–12, 14, 17, 18, 20, 21, 23, 26–28, 33, 35, 36, 39, 41–44) showed that the procedure time was higher in the GA group (Figure 5), with a statistically significant difference ((MD = 20.22, 95%CI, 15.37–25.07, *P* < 0.00001). The subgroup analysis showed that the procedure time was significantly lower in the CS subgroup (MD = 11.97, 95%CI, 9.19–14.75, *P* < 0.00001), LA subgroup (MD = 27.96, 95%CI, 7.37–48.54, *P* = 0.008), and MAC subgroup (MD = 23.70, 95%CI, 10.10–37.31, *P* = 0.0006).

#### Length of hospital stay and ICU stay

**Figure 6:**
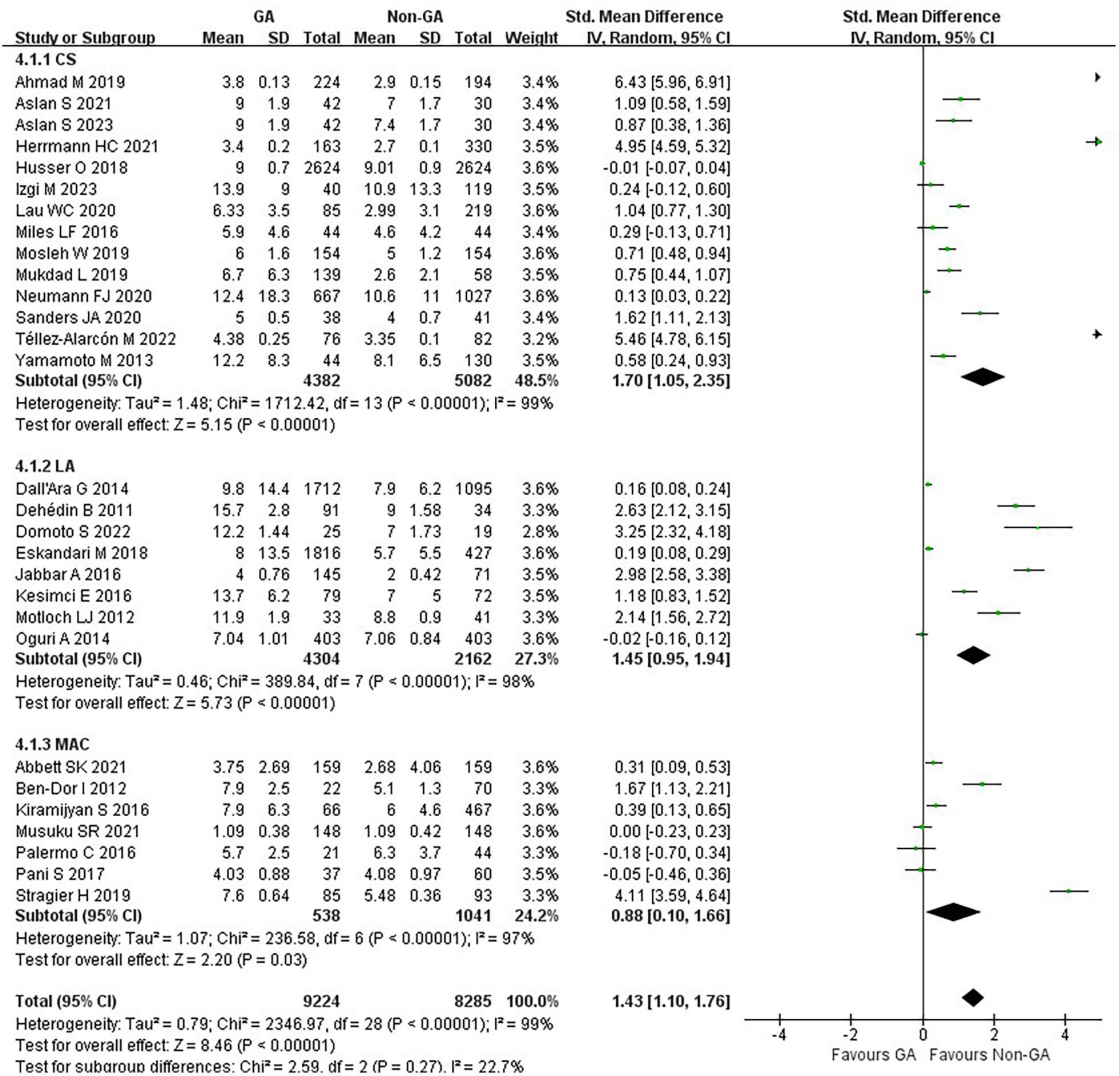
Meta-analysis of the length of stay hospital.

Length of hospital stay based on 29 studies (8–12, 14, 15, 17–23, 26–29, 31, 33, 36–43) showed that the length of hospital stay in the GA group was higher than that in the control group (Figure 6), with a statistically significant difference (MD = 1.43, 95%CI, 1.10–1.76, *P* < 0.00001). The subgroup analysis showed that the length of hospital stay in the CS subgroup (MD = 1.70, 95%CI, 1.05–2.35, *P* < 0.00001), LA subgroup (MD = 1.45, 95%CI, 0.95–1.94, *P* < 0.00001), and MAC subgroup (MD = 0.88, 95%CI, 0.10–1.66, *P* = 0.03) were significantly lower.

**Figure 7:**
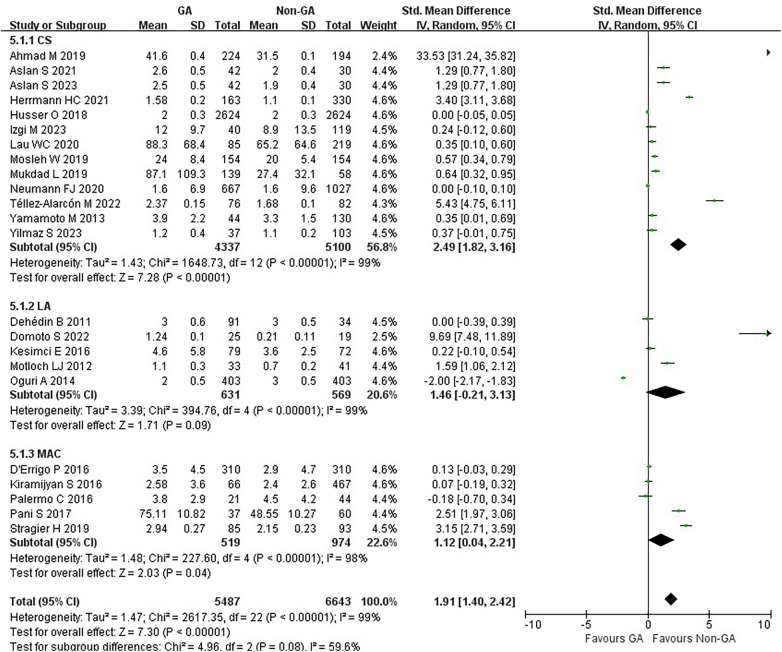
Meta-analysis of the ICU stay.

Based on 30 studies (8, 10, 12–15, 17, 19, 23, 28, 29, 31, 33, 36–44), the ICU stay in the GA group was longer than that of other anesthetic methods (SMD = 1.91, 95%CI, 1.40–2.42, *P* < 0.00001) (Figure 7). The subgroup analysis showed that the ICU stay in the CS subgroup (SMD = 2.49, 95%CI, 1.83–3.16, *P* < 0.00001) and MAC subgroup (SMD = 1.12, 95%CI, 0.04–2.21, *P* = 0.04) was lower, with a statistically significant difference. However, there was no significant difference in ICU stay between the GA group and LA subgroup (SMD = 1.46; 95%CI, −0.21–3.13, *P* = 0.09).

#### Postoperative acute kidney injury and stroke

**Figure 8:**
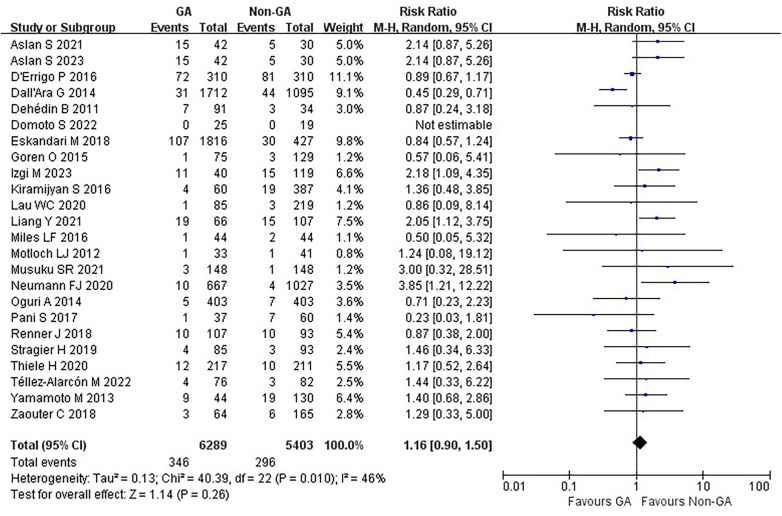
Meta-analysis of the incidence of acute kidney injury.

**Figure 9:**
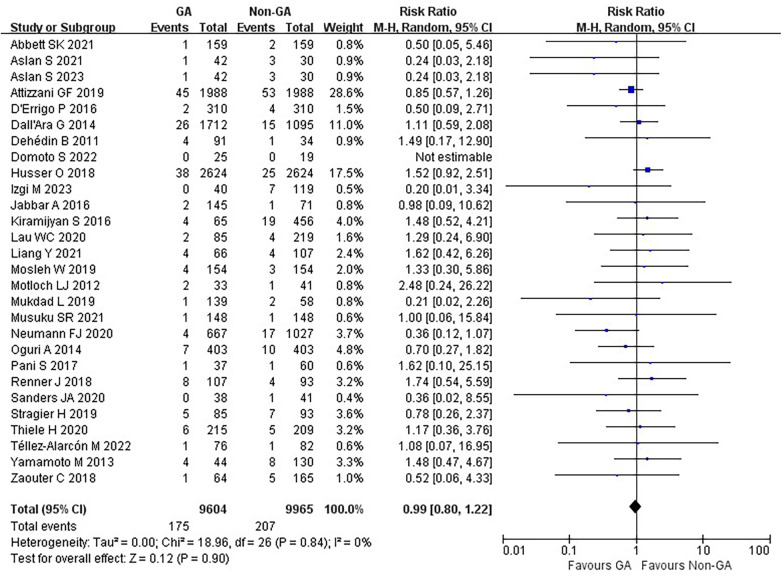
Meta-analysis of the incidence of stroke.

The incidence of postoperative acute kidney injury and stroke was reported in 24 studies (8, 11–17, 19–21, 23, 25, 26, 29, 30, 34, 35, 37–39, 42, 43) and 28 studies were included (8–19, 21–23, 25, 29, 30, 32, 33, 35, 37–40, 42, 43). However, there was no statistically significant difference in the incidence of postoperative acute kidney injury (RR = 1.16, 95%CI, 0.90–1.50, *P* = 0.26) and stroke (RR = 0.99, 95%CI, 0.80–1.22, *P* = 0.90), as shown in Figures 8, 9.

#### Postoperative vascular complications and myocardial infarction

**Figure 10:**
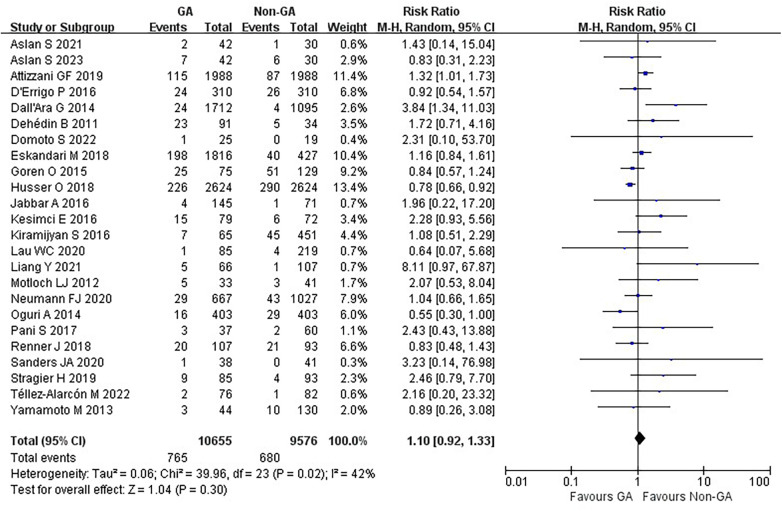
Meta-analysis of the incidence of vascular complication.

**Figure 11:**
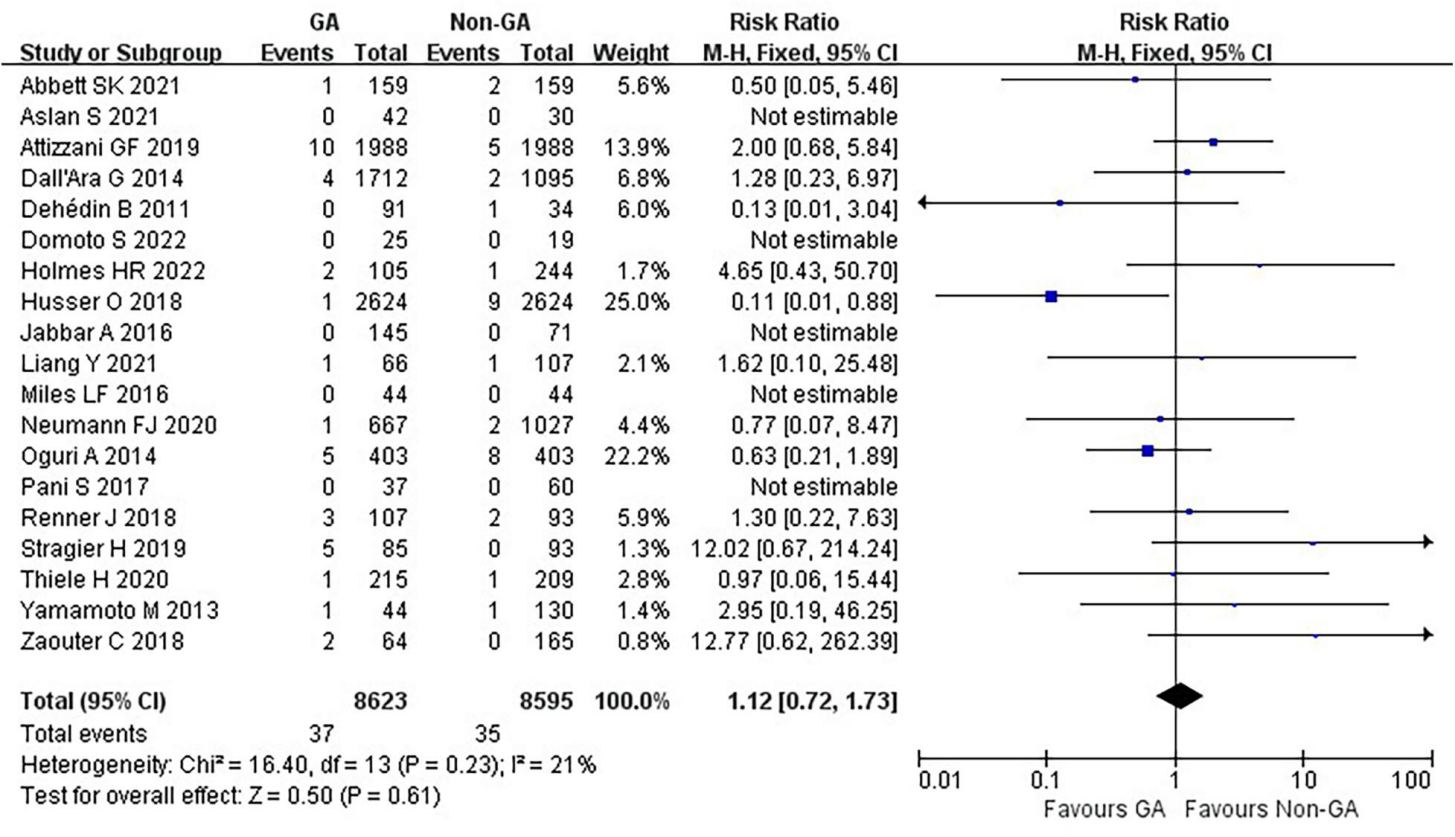
Meta-analysis of the incidence of myocardial infarction.

The incidence of postoperative vascular complications and myocardial infarction was reported in 24 studies (8–20, 23, 29, 32, 34–39, 42) and 19 studies (8, 10–12, 14–16, 18, 19, 22–26, 29, 30, 32, 35, 42) respectively. Moreover, the difference in postoperative vascular complications (RR = 1.10, 95%CI, 0.92–1.33, *P* = 0.30) (Figure 10) and the difference of postoperative myocardial infarction (RR = 1.12, 95%CI, 0.72–1.73, *P* = 0.61) was not statistically significant (Figure 11).

#### Postoperative pneumonia

**Figure 12:**
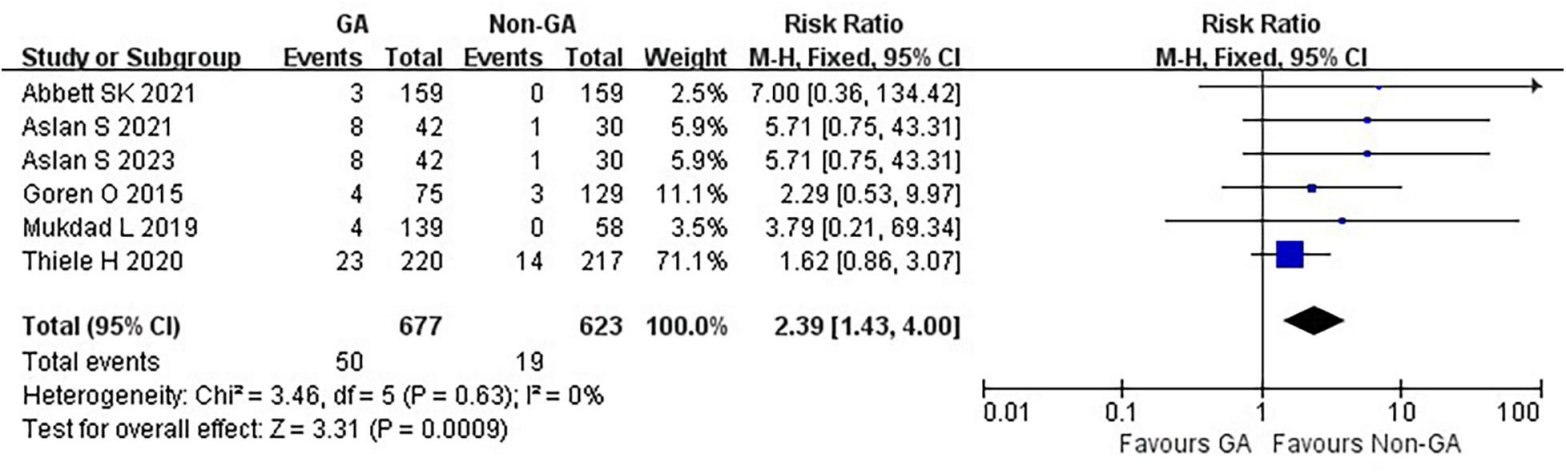
Meta-analysis of the incidence of pneumonia.

The incidence of postoperative pneumonia in the GA group was higher than that in the control group based on six studies (22, 23, 30, 33, 34). The difference was statistically significant (RR = 2.39, 95%CI, 1.43–4.00, *P* = 0.0009), as shown in Figure 12.

### Publication bias analysis

**Figure 13:**
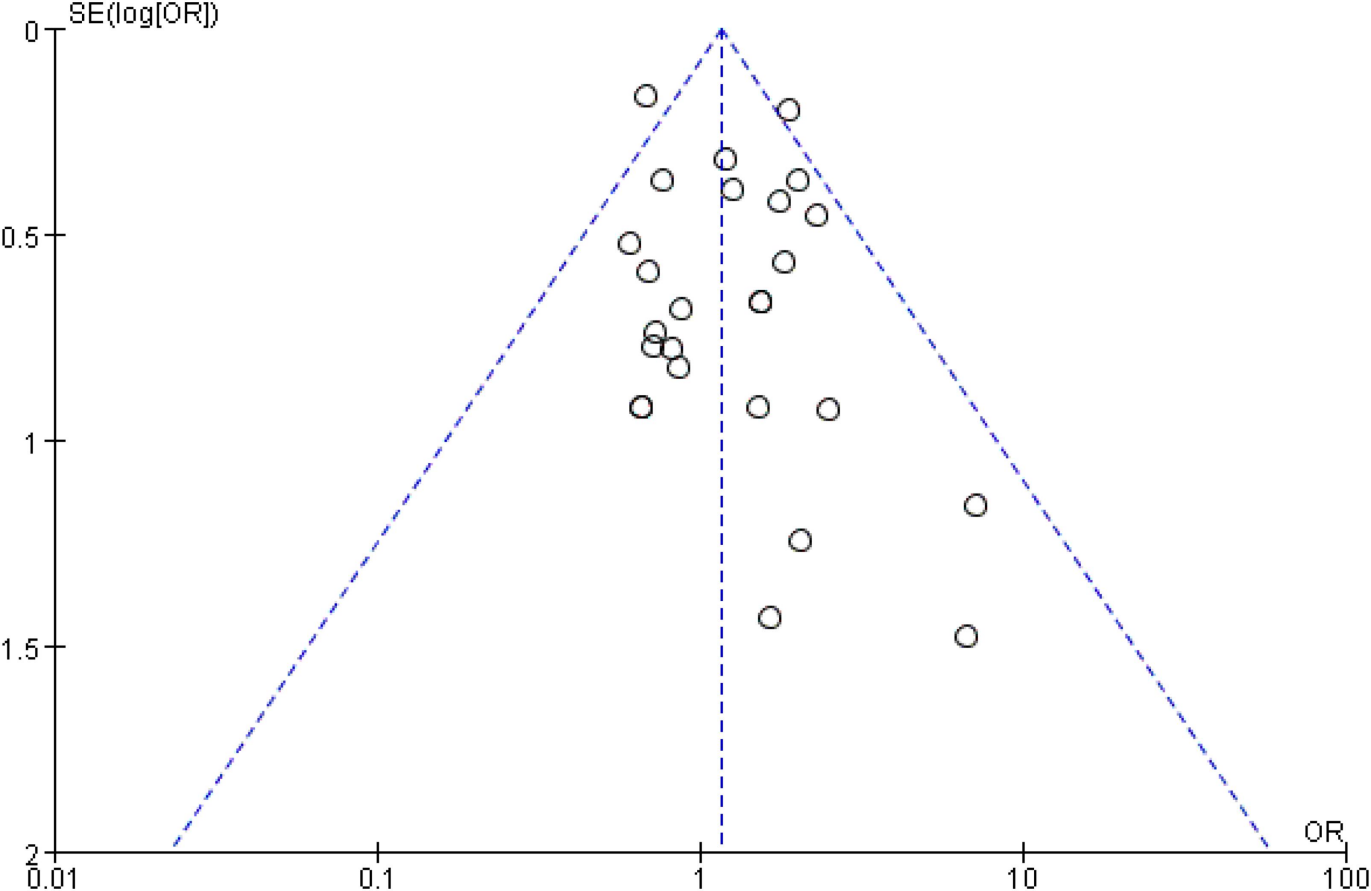
Funnel plot of the day 30 mortality.

The funnel plot of 30-day mortality in the included studies was symmetrical, resulting in a small publication bias, as shown in Figure 13.

## Discussion

The present systematic review and meta-analysis analyzed the impact of general anesthesia and other non-general anesthesia approaches on the complications and prognosis of patients undergoing TAVI. We found that general anesthesia extended the procedure time, prolonged the hospital and ICU stay, and increased the incidence of postoperative pneumonia and in-hospital mortality. Nevertheless, compared with non-general anesthesia, general anesthesia does not increase the incidence of postoperative complications, such as acute kidney injury, stroke, myocardial infarction, and vascular complications, and it does not increase the long-term quality of life. These results were consistent with those of recently published systemic reviews, which concluded that TAVI with local anesthesia was associated with a significant reduction in-hospital stay (45). However, we found that general anesthesia did not increase day 30 mortality, which differs from the results of some previous studies (45). However, considering that in addition to local anesthesia, there are still many types of anesthetic approaches used in the non-GA group, ranging from local lidocaine infiltration of the access sites to different degrees of sedation, we not only compared general anesthesia and local anesthesia, but also observed the effects of other anesthesia methods, such as MAC and conscious/deep sedation, on TAVI. Of course, we should note the unbalanced distribution of anesthetic approaches over the study period. Overall, our study adds new information to the decision-making regarding the optimal anesthetic strategy for TAVI.

An increasing number of reviews have discussed TAVI surgery performed traditionally under general anesthesia or other anesthesia methods (including RA, LA, MAC, CS et al.). One of the reasons for this is the advancement of surgical technology and the expansion of surgical indications for younger Patients (46) with less dependence on transesophageal echocardiography (TEE), new types of easy-manipulate valves, and smaller sheath sizes. Other procedural approaches, including transapical, transaortic, and transcaval TAVI, are usually considered unsuitable for Non-GA approaches (9, 47). Another reason is that whether SAVR or TAVI, patients may experience acute kidney injury, stroke, myocardial infarction, and vascular-related complications after surgery, and their postoperative survival and quality of life may be affected by different anesthetic modalities. Anesthesia management may induce different pathophysiological changes in the organism, which can also affect postoperative survival and related adverse complications in patients. Therefore, the choice of anesthesia should be optimized during TAVI to minimize the incidence of postoperative complications and improve long-term quality of life.

However, because of the paucity of randomized clinical trial data and the fact that the findings are mainly based on retrospective studies, the outcomes may be susceptible to selection bias. Although the most frequently considered rationale for preferring general anesthesia is the increased convenience of handling some sudden life-threatening emergencies during TAVI, such as coronary obstruction, aortic dissection, and valve embolization, most studies agreed that compared with general anesthesia, RA, LA, MAC, and other non-general anesthesia methods had shorter procedure time, thus decreasing hospital and ICU stay, lower postoperative pneumonia, and in-hospital mortality, which may benefit from increased hemodynamic stability with non-general anesthesia and minimize the cardiovascular and postoperative-pulmonary complications associated with general anesthesia (8, 48, 49). Meanwhile, to ensure safety, the surgeon may speed up the procedure to relieve the patient's tension. Our results also showed that patients in the GA group had higher in-hospital mortality rates than those in the non-GA group. The results of the subgroup analysis further showed that in-hospital mortality was lower in the CS subgroup than in the LA subgroup. Interestingly, the subgroup analysis in our study found no difference in in-hospital mortality between the MAC and GA groups, unlike previous studies. However, the different anesthetic modalities did not affect 30-day postoperative mortality, which is in accordance with the results of previous related studies (18). Therefore, although GA increases postoperative in-hospital mortality compared with non-general anesthesia, it does not increase the risk of long-term mortality of patient. The possible reason is that patients undergoing TAVI surgery often have a greater number of underlying diseases, resulting in poor long-term quality of life and a lower survival rate. The pathological and physiological changes brought about by anesthesia methods mainly affect the short-term postoperative mortality rate and do not pose a threat to the long-term mortality rate of patients.

We also found that patients in the GA group had a higher incidence of postoperative pneumonia. However, the results of the subgroup analysis showed no difference in ICU admission time between the GA and non-GA groups, in contrast to the findings of previous studies (50). This may be because the prognosis of TAVI surgery is more related to the surgical site, procedural experience, surgical preference, etc. Therefore, compared with non-GA modalities, general anesthesia increases the incidence of postoperative pneumonia in patients, thus further prolonging the length of hospital stay, and needs to be chosen with caution. However, whether general anesthesia increases the duration of ICU stay requires further studies, as the duration of ICU stay is influenced by a variety of complex procedural factors. The results of our study showed no difference in the incidence of stroke, myocardial infarction, and vascular complications between the general anesthesia group and the control group, in contrast to the views of Brecker, who conducted a multicenter advance study and showed that more common major vascular complications occurred in the non-GA groups. The total hospital stay was similar between the two groups (51), which may be because non-GA patients have more tension due to wakefulness and restlessness. In particular, it is very helpful to ensure that the patient is absolutely immobile when the prosthetic valve is deployed to the optimal position. The randomized SOLVE-TAVI trial conducted by Feistritzer (52) also showed that the combined endpoint of all-cause mortality occurred with similar rates between the groups.

There are still differing opinions regarding the incidence of AKI. Some studies found an increased risk of acute kidney injury due to LA in patients undergoing TAVI (43, 53), which is inconsistent with our results. During general anesthesia, there may be more fluid input to ensure hemodynamic stability, which may be one of the reasons for the decreased acute kidney injury. Furthermore, GA can better relieve pain and alleviate anxiety by placing the patient in a state of “unconsciousness.” Further RCTs are required to verify the most likely favorable anesthetic approach for TAVI.

This study had some limitations. The gray literature was not searched or screened. The heterogeneity in anesthetic techniques, differences in TAVI devices, and procedural evolution over time all constitute potential confounding variables in this study, which to some extent increase the heterogeneity of the results. In addition, the effects of operator and center learning-curves also increased the heterogeneity of the results. Therefore, a large number of multicenter randomized controlled trials with large sample sizes and rigorous designs are needed to provide a stronger scientific basis for clinical practice.

## Conclusion

General anesthesia increases in-hospital mortality and the incidence of postoperative pulmonary infections in patients undergoing TAVI compared with many non-general anesthesia methods (including RA, LA, and CS), But MAC under non-general anesthesia did not reduce in-hospital mortality. GA prolongs length of hospitalization and ICU stay. However, it did not increase the incidence of postoperative acute kidney injury, stroke, myocardial infarction, or vascular complications in patients and did not increase the 30-day postoperative mortality rate or the long-term quality of patients. Therefore, the choice of anesthesia for TAVI should be evaluated according to the patient's condition and surgical approach to minimize adverse complications and mortality.

## References

[1] Leon MB Smith CR Mack M Miller DC Moses JW Svensson LG. Transcatheter aortic-valve implantation for aortic stenosis in patients who cannot undergo surgery. N Engl J Med. (2010) 363(17):1597–607. 10.1056/NEJMoa1008232 20961243

[2] Mäkikallio T Jalava MP Husso A Virtanen M Laakso T Ahvenvaara T. Ten-year experience with transcatheter and surgical aortic valve replacement in Finland. Ann Med. (2019) 51(3–4):270–9. 10.1080/07853890.2019.1614657 31112060 PMC7880078

[3] Terkelsen CJ Thim T Freeman P Dahl JS Nørgaard BL Kim WY. Randomized comparison of TAVI valves: the compare-TAVI trial. Am Heart J. (2024) 274:84–94. 10.1016/j.ahj.2024.05.003 38729550

[4] Rex S. Anesthesia for transcatheter aortic valve implantation: an update. Curr Opin Anaesthesiol. (2013) 26:456–66. 10.1097/ACO.0b013e3283628d1e 23743555

[5] Anderson RD Gargus N Randall MH. Editorial: the use of fascia iliaca block with minimal conscious sedation in transcatheter aortic valve replacement: advances in TAVR anesthesia. Cardiovasc Revasc Med. (2020) 21:602–3. 10.1016/j.carrev.2020.03.017 32201210

[6] Cumpston M Li T Page MJ Chandler J Welch VA Higgins JP. Updated guidance for trusted systematic reviews: a new edition of the Cochrane handbook for systematic reviews of interventions. Cochrane Database Syst Rev. (2019) 10:ED000142. 10.1002/14651858.ED000142 31643080 PMC10284251

[7] Li G Yu L Yang Y Deng J Shao L Zeng C. Effects of perioperative music therapy on patients with postoperative pain and anxiety: a systematic review and meta-analysis. J Integr Complement Med. (2024) 30(1):37–46. 10.1089/jicm.2022.0803 37646752

[8] Domoto S Nakazawa K Yamaguchi J Hayakawa M Otsuki H Inagaki Y. Minimum-incision trans-subclavian transcatheter aortic valve replacement with regional anesthesia. J Cardiol. (2023) 81:131–7. 10.1016/j.jjcc.2022.07.006 35882612

[9] Sanders JA Vaidyanathan A Sayeed H Sherdiwala B Wyman J Wang DD. Comparison of deep sedation and general anesthesia with an endotracheal tube for transcaval transcatheter aortic valve replacement: a pioneering institution’s experience. J Cardiothorac Vasc Anesth. (2021) 35:2607–12. 10.1053/j.jvca.2020.12.031 33441271

[10] Husser O Fujita B Hengstenberg C Frerker C Beckmann A Möllmann H. Conscious sedation versus general anesthesia in transcatheter aortic valve replacement: the German aortic valve registry. JACC Cardiovasc Interv. (2018) 11:567–78. 10.1016/j.jcin.2017.12.019 29566803

[11] Butala NM Chung M Secemsky EA Manandhar P Marquis-Gravel G Kosinski AS. Conscious sedation versus general anesthesia for transcatheter aortic valve replacement: variation in practice and outcomes. JACC Cardiovasc Interv. (2020) 13:1277–87. 10.1016/j.jcin.2020.03.008 32499018 PMC7650030

[12] Dehedin B Guinot PG Ibrahim H Dehédin B Allou N Provenchère S. Anesthesia and perioperative management of patients who undergo transfemoral transcatheter aortic valve implantation: an observational study of general versus local/regional anesthesia in 125 consecutive patients. J Cardiothorac Vasc Anesth. (2011) 25:1036–43. 10.1053/j.jvca.2011.05.008 21803602

[13] D'Errigo P Ranucci M Covello RD D’Errigo P Biancari F Rosato S. Outcome after general anesthesia versus monitored anesthesia care in transfemoral transcatheter aortic valve replacement. J Cardiothorac Vasc Anesth. (2016) 30:1238–43. 10.1053/j.jvca.2016.05.034 27495961

[14] Stragier H Dubois C Verbrugghe P Jacobs S Adriaenssens T Rex S. General anesthesia versus monitored anesthesia care for transfemoral transcatheter aortic valve implantation: a retrospective study in a single Belgian referral center. J Cardiothorac Vasc Anesth. (2019) 33:3283–91. 10.1053/j.jvca.2019.06.027 31350148

[15] Neumann FJ Redwood S Abdel-Wahab M Lefèvre T Frank D Eltchaninoff H. General anesthesia or conscious sedation for transfemoral aortic valve replacement with the SAPIEN 3 transcatheter heart valve. Int Heart J. (2020) 61:713–9. 10.1536/ihj.19-567 32684591

[16] Liang Y Wang W Wang X Liu M Hei F Guan Y. General anesthesia increased the risk of atrial fibrillation and acute kidney injury in transcatheter aortic valve replacement. Heart Surg Forum. (2021) 24:E082–100. 10.1532/hsf.3361 33635259

[17] Motloch LJ Rottlaender D Reda S Larbig R Bruns M Müller-Ehmsen J. Local versus general anesthesia for transfemoral aortic valve implantation. Clin Res Cardiol. (2012) 101:45–53. 10.1007/s00392-011-0362-8 21931964

[18] Jabbar A Khurana A Mohammed A Das R Zaman A Edwards R. Local versus general anesthesia in transcatheter aortic valve replacement. Am J Cardiol. (2016) 118:1712–6. 10.1016/j.amjcard.2016.08.051 27692595

[19] Pani S Cagino J Feustel P Musuku SR Raja A Bruno N. Patient selection and outcomes of transfemoral transcatheter aortic valve replacement performed with monitored anesthesia care versus general anesthesia. J Cardiothorac Vasc Anesth. (2017) 31:2049–54. 10.1053/j.jvca.2017.04.005 28911896

[20] Eskandari M Aldalati O Dworakowski R Byrne JA Alcock E Wendler O. Comparison of general anaesthesia and non-general anaesthesia approach in transfemoral transcatheter aortic valve implantation. Heart. (2018) 104:1621–8. 10.1136/heartjnl-2017-312559 29599379

[21] Musuku SR Capua CAD Doshi I Cherukupalli D Byun Y Shapeton AD. Outcomes of transfemoral transcatheter aortic valve replacement performed with general anesthesia using a supraglottic airway versus monitored anesthesia care. J Cardiothorac Vasc Anesth. (2021) 35:1760–8. 10.1053/j.jvca.2020.09.086 32980257

[22] Abbett SK Urman RD Resor CD Brovman EY. The effect of anesthesia type on outcomes in patients undergoing transcatheter aortic valve replacement. J Cardiothorac Vasc Anesth. (2021) 35:429–35. 10.1053/j.jvca.2020.09.083 33023815

[23] Aslan S Güner A Demir AR Yılmaz E Aslan AF Çelik Ö Uzun F Ertürk M Conscious sedation versus general anesthesia for transcatheter aortic valve implantation in patients with severe chronic obstructive pulmonary disease. Perfusion. (2023) 38:186–92. 10.1177/02676591211045801 34590527

[24] Holmes HR Falasa M Neal D Choi CY Park K Bavry AA. Monitored anesthesia care versus general anesthesia for transcatheter aortic valve replacement. Innovations. (2022) 17:401–8. 10.1177/15569845221124113 36217748

[25] Zaouter C Smaili S Leroux L Bonnet G Leuillet S Ouattara A. Transcatheter aortic valve implantation: general anesthesia using transesophageal echocardiography does not decrease the incidence of paravalvular leaks compared to sedation alone. Ann Card Anaesth. (2018) 21:277–84. 10.4103/aca.ACA_204_17 30052215 PMC6078031

[26] Miles LF Joshi KR Ogilvie EH Densem CG Klein AA O’Sullivan M. General anaesthesia vs. conscious sedation for transfemoral aortic valve implantation: a single UK centre before-and-after study. Anaesthesia. (2016) 71:892–900. 10.1111/anae.13522 27353456

[27] Ben-Dor I Looser PM Maluenda G Weddington TC Kambouris NG Barbash IM. Transcatheter aortic valve replacement under monitored anesthesia care versus general anesthesia with intubation. Cardiovasc Revasc Med. (2012) 13:207–10. 10.1016/j.carrev.2012.02.002 22818531

[28] Herrmann HC Cohen DJ Hahn RT Babaliaros VC Yu X Makkar R. Utilization, costs, and outcomes of conscious sedation versus general anesthesia for transcatheter aortic valve replacement. Circ Cardiovasc Interv. (2021) 14:e010310. 10.1161/CIRCINTERVENTIONS.120.010310 34130476

[29] Oguri A Yamamoto M Mouillet G Gilard M Laskar M Eltchaninoff H. Clinical outcomes and safety of transfemoral aortic valve implantation under general versus local anesthesia: subanalysis of the French aortic national core valve and Edwards 2 registry. Circ Cardiovasc Interv. (2014) 7:602–10. 10.1161/CIRCINTERVENTIONS.113.000403 25006175

[30] Thiele H Kurz T Feistritzer HJ Stachel G Hartung P Lurz P. General versus local anesthesia with conscious sedation in transcatheter aortic valve implantation: the randomized SOLVE-TAVI trial. Circulation. (2020) 142:1437–47. 10.1161/CIRCULATIONAHA.120.046451 32819145

[31] Ahmad M Patel JN Vipparthy SC Divecha C Barzallo PX Kim M. Conscious sedation versus general anesthesia in transcatheter aortic valve replacement: a cost and outcome analysis. Cureus. (2019) 11:e4812. 10.7759/cureus.4812 31281765 PMC6599466

[32] Attizzani GF Patel SM Dangas GD Szeto WY Sorajja P Reardon MJ. Comparison of local versus general anesthesia following transfemoral transcatheter self-expanding aortic valve implantation (from the transcatheter valve therapeutics registry). Am J Cardiol. (2019) 123:419–25. 10.1016/j.amjcard.2018.10.041 30527797

[33] Mukdad L Kashani R Mantha A Sareh S Mendelsohn A Benharash P. The incidence of dysphagia among patients undergoing TAVR with either general anesthesia or moderate sedation. J Cardiothorac Vasc Anesth. (2019) 33:45–50. 10.1053/j.jvca.2018.05.040 30057252

[34] Goren O Finkelstein A Gluch A Sheinberg N Dery E Matot I. Sedation or general anesthesia for patients undergoing transcatheter aortic valve implantation–does it affect outcome? An observational single-center study. J Clin Anesth. (2015) 27:385–90. 10.1016/j.jclinane.2015.03.025 25912486

[35] Renner J Tesdorpf A Freitag-Wolf S Freitag-Wolf S Francksen H Petzina R. A retrospective study of conscious sedation versus general anaesthesia in patients scheduled for transfemoral aortic valve implantation: a single center experience. Health Sci Rep. (2019) 2:e95. 10.1002/hsr2.95 30697594 PMC6346987

[36] Kesimci E Erkiliç E Gümüş T Kanbak O. Impact of different anesthetic managements in outcomes of transcatheteraortic valve implantation: the first Turkish experience. Turk J Med Sci. (2016) 46:742–8. 10.3906/sag-1503-74 27513250

[37] Kiramijyan S Ben-Dor I Koifman E Didier R Magalhaes MA Escarcega RO. Comparison of clinical outcomes with the utilization of monitored anesthesia care vs. general anesthesia in patients undergoing transcatheter aortic valve replacement. Cardiovasc Revasc Med. (2016) 17:384–90. 10.1016/j.carrev.2016.02.003 27133500

[38] Lau WC Shannon FL Hanzel GS Safian RD Abbas AE Sakwa MP. Transfemoral transcatheter aortic valve replacement using fascia iliaca block as an alternative approach to conscious sedation as compared to general anesthesia. Cardiovasc Revasc Med. (2020) 21:594–601. 10.1016/j.carrev.2019.08.080 31523003

[39] Tellez-Alarcon M Montes FR Hurtado P Gutiérrez LP Cabrales JR Camacho J. Conscious sedation versus general anesthesia for transcatheter aortic valve implantation: a retrospective study. Braz J Anesthesiol. (2022) 72:539–41. 10.1016/j.bjane.2021.11.009 34929219 PMC9373643

[40] Mosleh W Mather JF Amer MR Hiendlmayr B Kiernan FJ McKay RG. Propensity matched analysis comparing conscious sedation versus general anesthesia in transcatheter aortic valve implantation. Am J Cardiol. (2019) 124:70–7. 10.1016/j.amjcard.2019.03.042 31064667

[41] Palermo C Degnan M Candiotti K Salerno T de Marchena E Rodriguez-Blanco Y. Monitored anesthesia care versus general anesthesia: experience with the medtronic CoreValve. J Cardiothorac Vasc Anesth. (2016) 30:1234–7. 10.1053/j.jvca.2016.02.006 27222049

[42] Yamamoto M Meguro K Mouillet G Bergoend E Monin J-L Lim P. Effect of local anesthetic management with conscious sedation in patients undergoing transcatheter aortic valve implantation. Am J Cardiol. (2013) 111:94–9. 10.1016/j.amjcard.2012.08.053 23068861

[43] Izgi M Halis A Şener YZ Şahiner L Kaya EB Aytemir K. Evaluation of anaesthetic approaches in transcatheter aortic valv implantation procedures. Turk J Anaesthesiol Reanim. (2023) 51(5):427–33. 10.4274/TJAR.231270 37876170 PMC10606737

[44] Yılmaz Ş Zeren G Avcı İİ Sungur MA Can F Yılmaz MF. The effect of anesthesia type applied in transcatheter aortic valve implantation. Turk Kardiyol Dern Ars. (2023) 51(6):394–8. 10.5543/tkda.2023.38920 37671519

[45] Wang L Liu Y Gao H Zhang B Zhou S Xie M. Comparison of safety and effectiveness of local or general anesthesia after transcatheter aortic valve implantation: a systematic review and meta-analysis. J Clin Med. (2023) 12:508. 10.3390/jcm12020508 36675437 PMC9866516

[46] Mach M Poschner T Hasan W Kerbel T Szalkiewicz P Hasimbegovic E. Transcatheter versus isolated surgical aortic valve replacement in young high-risk patients: a propensity score-matched analysis. J Clin Med. (2021) 10:3447. 10.3390/jcm10153447 34362230 PMC8346998

[47] Feistritzer HJ Ender J Lauten P Rudolph TK Rudolph V Geisler T. DOUBLE-CHOICE Investigators. Peri-interventional anesthesia strategies for transcatheter aortic valve implantation: a multicenter, randomized, controlled, noninferiority trial. Circulation. (2025) 152(22):1526–37. 10.1161/CIRCULATIONAHA.125.076557 40878766

[48] Gao L Jin B Chao C Wang B Zhang X Shen J. Comparative efficacy of local and general anesthesia for transcatheter aortic valve implantation: a meta-analysis and systematic review. Heart Surg Forum. (2022) 25:E364–73. 10.1532/hsf.4631 35787764

[49] Frohlich GM Lansky AJ Webb J Roffi M Toggweiler S Reinthaler M. Local versus general anesthesia for transcatheter aortic valve implantation (TAVR)–systematic review and meta-analysis. BMC Med. (2014) 12:41. 10.1186/1741-7015-12-41 24612945 PMC4022332

[50] Maldonado Y Baisden J Villablanca PA Weiner MM Ramakrishna H. General anesthesia versus conscious sedation for transcatheter aortic valve replacement-an analysis of current outcome data. J Cardiothorac Vasc Anesth. (2018) 32:1081–6. 10.1053/j.jvca.2017.08.006 29338998

[51] Brecker SJ Bleiziffer S Bosmans J Gerckens U Tamburino C Wenaweser P. Impact of anesthesia type on outcomes of transcatheter aortic valve implantation (from the multicenter ADVANCE study). Am J Cardiol. (2016) 117:1332–8. 10.1016/j.amjcard.2016.01.027 26892451

[52] Feistritzer HJ Kurz T Stachel G Hartung P Lurz P Eitel I. Impact of anesthesia strategy and valve type on clinical outcomes after transcatheter aortic valve replacement. J Am Coll Cardiol. (2021) 77:2204–15. 10.1016/j.jacc.2021.03.007 33926657

[53] Ehret C Rossaint R Foldenauer AC Stoppe C Stevanovic A Dohms K. Is local anaesthesia a favourable approach for transcatheter aortic valve implantation? A systematic review and meta-analysis comparing local and general anaesthesia. BMJ Open. (2017) 7:e016321. 10.1136/bmjopen-2017-016321 28951409 PMC5623571

